# Experimental and Monte Carlo simulation study on a core–shell NiFe_2_O_4_@HKUST-1/graphene oxide nanocomposite for Congo Red adsorption†

**DOI:** 10.1039/d5ra02381e

**Published:** 2025-07-02

**Authors:** Edris Jamshidi, Maryam Haddadi, Faranak Manteghi, Rahime Eshaghi Malekshah, Zari Tehrani

**Affiliations:** a Research Laboratory of Inorganic Chemistry and Environment, Department of Chemistry, Iran University of Science and Technology Tehran 1684613114 Iran f_manteghi@iust.ac.ir; b Department of Medicinal and Applied Chemistry, Drug Development and Value Creation Research Centre, Kaohsiung Medical University Kaohsiung 807 Taiwan; c Department of Chemistry, Semnan University Semnan 35131-19111 Iran; d The Future Manufacturing Research Institute, Faculty of Science and Engineering, Swansea University SA1 8EN Swansea UK z.tehrani@swansea.ac.uk

## Abstract

A copper-based metal–organic framework, nickel ferrite and graphene oxide were prepared as constituents of a new core–shell nanocomposite formed by a layer-by-layer method, then it was applied to absorb Congo Red dye as an organic contaminant. The nanocomposite was studied by XRD, FTIR, EDS, FESEM and VSM methods. Investigating the main factors affecting the adsorption shows that the optimum pH of the dye solution is 7, the best contact time is 60 min with an initial solution concentration of 5 ppm and 0.05 g of adsorbent is the optimum amount. Adaptation of Langmuir, Freundlich, Temkin and Dubinin–Radushkevich adsorption isotherms showed that the dye adsorption process is consistent with two first isotherm models. Regarding the adsorption kinetics and according to the calculations, it was found that the adsorption process follows second-order kinetics. The composite NiFe_2_O_4_@HKUST-1/GO demonstrated a maximum adsorption capacity of 25.64 mg g^−1^ for Congo Red dye removal from aqueous solutions. Monte Carlo simulations were used to simulate the adsorption nature between NiFe_2_O_4_ (311) molecules and the HKUST-1 surface, GO molecules and NiFe_2_O_4_@HKUST-1, and CR and NiFe_2_O_4_@HKUST-1/GO.

## Abstract 2

A novel core–shell NiFe_2_O_4_@HKUST-1/GO nanocomposite was synthesized for enhanced dye adsorption, combining experimental characterization with Monte Carlo simulations.

## Graphical abstract

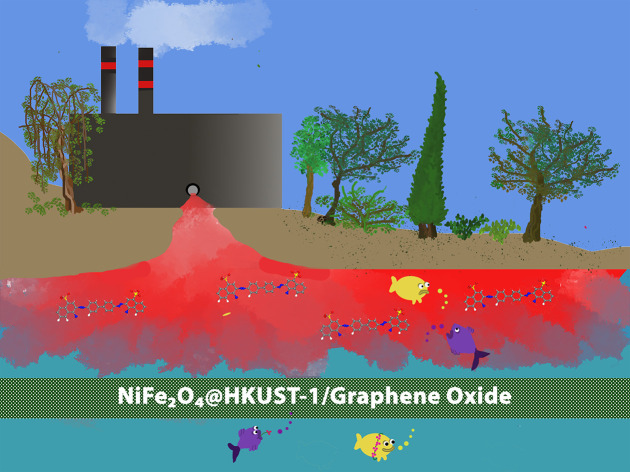

## 1. Introduction

Dyes, despite their aesthetic and visual appeal, are among the most serious pollutants in wastewater.^1,2^ Their extensive use in various industrial applications, such as cosmetics, textiles, and the food industry, cannot be overlooked.^3^ Among many kinds of dyes, organic dyes are highly toxic, since they contain one or more aromatic rings. However, for this reason, they are stable and resistant to degradation.^4^ During the dyeing processes in these industries, wastewater containing dyes is discharged into water bodies, posing significant hazards due to their potential mutagenic and carcinogenic effects on both human health and aquatic life.^5–8^ Azo dyes represent the largest class of dyes, offering the widest variety of colors.^9,10^ However, they are resistant to degradation under aerobic conditions. Among these, Congo Red, an azo anionic dye that is water-soluble, poses significant health risks.^11^ According to its Safety Data Sheet (SDS), Congo Red is classified as a potential carcinogen and is suspected of causing harm to unborn children. Although Congo Red was historically used extensively in cotton dyeing, it has been largely replaced by dyes that offer greater resistance to light and washing. However, its intense coloration poses a significant environmental threat.^12^ By reducing sunlight penetration into aquatic ecosystems, Congo Red disrupts photosynthetic processes, leading to irreversible damage to marine life and the broader ecosystem.^13,14^ The degradation of toxic dyestuffs is particularly challenging due to their high stability against light and oxidants, making them persistent and harmful pollutants with a significant environmental impact.^15^

Therefore, their removal from wastewater became a global concern.^16,17^ Various methods are available for removing this type of pollution from wastewater, including biological treatment, reverse osmosis, nanofiltration, coagulation, chemical oxidation, sedimentation, and adsorption, among others.^18,19^ Among the methods, adsorption has become the most remarkable technology in recent decades.^19^ Adsorption means transferring mass between two different or similar phases, for example, liquid–liquid, liquid–solid, gas–liquid, and gas–solid.^20^ An adsorbent is a porous, insoluble material capable of capturing and trapping adsorbate particles on its surface. However, the high cost of some adsorbents has prompted researchers to explore and develop low-cost alternatives, which have been extensively documented in numerous research studies.^21^

Active carbon as one of the traditional adsorbents usually applied for removing the dyestuffs from wastewater, but the most important disadvantage of active carbon is that it acts efficiently in low concentration of dyes.^22^ Among the different kinds of adsorbent (active-carbon, zeolites, *etc.*), the metal–organic-frameworks (MOFs) are a breathtaking class of porous materials with lots of exclusive features, such as tunable pore size, high chemical, and physical stability, high specific surface.^23,24^ Metal–Organic Frameworks (MOFs) play a versatile functional role in various applications, including sensing, gas storage, ion exchange, adsorption, catalysis, separation, and many other areas.^25–27^ MOFs are crystalline nanoporous materials composed of metal oxide clusters coordinated with organic linkers, forming three-dimensional networks.^28^ Some MOFs have been utilized for removing organic pollutants, particularly dyes, from aqueous solutions.^29,30^ Among these, HKUST-1 (Cu_3_(benzene-1,3,5-tricarboxylate)_2_) stands out as a copper-based MOF with a molecular formula of C_18_H_6_Cu_3_O_12_ and a molecular weight of 604.87 g mol^−1^. This MOF has garnered significant attention due to its high surface area, remarkable chemical stability, and accessible coordinatively unsaturated sites (CUS).^31^

Graphene oxide (GO) contains a variety of functional groups that make it suitable for constructing graphene-based hybrid composites.^32^ Graphene has high chemical stability and low reactivity but can be functionalized through oxidation to form graphene oxide (GO), which contains oxygen-rich groups like hydroxyl, carboxyl, and epoxy. These groups make GO water-dispersible and provide active sites for attaching other molecules. As a result, graphene, GO, and their composites are widely used in fields such as energy, environment, and advanced materials.^33^ Due to the synergistic effect between MOFs and GO, the adsorption capacity is enhanced, and the composites are more stable compared to the parent materials.^34^ MOF/GO composites combine the advantageous characteristics of carbonaceous graphene surfaces with the high porosity and tunability of MOFs. As a result, MOF/GO composites are anticipated to be effective adsorbents for wastewater treatment.^35^

Applying MOF-containing composites has proven to be a highly effective process for the adsorption or degradation of various pollutant dyes or metallic ions, as demonstrated in several reported instances.^36–38^ On the other hand, magnetic microspheres have gained increasing attention in recent years due to their exceptional physicochemical properties and significant biomedical applications. Among these magnetic nanoparticles, Fe_3_O_4_ – or more generally MFe_2_O_4_ (where M = Ni, Fe, Mn, Co) – has been considered a promising adsorbent for wastewater treatment.^39^ The advantages of magnetic MFe_2_O_4_ include low toxicity, ease of separation using external magnetic fields, the ability to agglomerate in solutions, and a high specific surface area.^40^ When effectively combined with MOFs, MFe_2_O_4_ can create MOF-based magnetic nanocomposites, which have the potential to remove a wide variety of pollutants from aqueous solutions.^41,42^

In recent research, there has been significant interest in the formation of core–shell magnetic materials, as their physical and chemical properties can be tuned by adjusting the core-to-shell size ratio, interface effects, and the attachment of various functional groups.^41^ In this study, a novel three-component nanocomposite consisting of a copper-based MOF, graphene oxide, and nickel ferrite has been synthesized in a core–shell configuration, characterized, and then applied for the removal of Congo Red (CR), an organic dye. The parameters affecting adsorption, such as pH, contact time, and varying concentrations of adsorbent and pollutant, were thoroughly investigated. Additionally, the study explores the sorption kinetics, isotherm models, and adsorption capacity of the composite. The characterization of the composite was conducted using a variety of techniques, including FESEM, XRD, FT-IR, VSM, BET, nitrogen adsorption–desorption, EDS, and UV-Vis.

## 2. Experimental

### 2.1. Materials and chemicals

The reactants used in this study include copper nitrate trihydrate (99%), benzene-1,3,5-tricarboxylic acid (H_3_BTC) (99%), anhydrous iron(iii) chloride (98%), nickel(ii) chloride (98%), sodium acetate(98%), mercaptoacetic acid (99%), graphene oxide functionalized with COOH group, ethylene glycol (99%), C_2_H_5_OH (98%) were all procured from Sigma-Aldrich (USA) and Merck (Germany) and used without further purification.

### 2.2. Instrumentation and measurements

Characterization of phases in all samples by X-ray diffraction (XRD) method in the range of 10–90° was established by D/max-IIIC (Shimadzu, Japan) with monochromatic CuKα radiation (*λ* = 1.5406 Å). Fourier Transform Infra-Red (FTIR) were taken with FT-IR Shimadzu (IR solution) 8400s, to identify the functional groups in the range of 4000–400 cm. Vibrating-sample magnetometry (VSM) was performed by using lakeshore (model 7407) instrument. Structure and morphological investigations were performed by Field Emission Scanning Electron Microscope (FESEM; Tescan Mira3) for iron-containing samples and scanning electron microscopy (SEM; Zeiss Supra ATM 55 acceleration 15k) for iron-free sample. The Brunauer–Emmett–Teller (BET) surface area measurement was analyzed by Micromeritics ASAP 2020 model. Energy dispersive X-ray (EDS; Tescan mira3) was used for the elemental analysis or chemical characterization of samples. Ultraviolet-visible spectroscopy (UV-Vis) was used for concentration measurement of dye in different conditions.

### 2.3. Methods

#### 2.3.1. Preparation of HKUST-1

HKUST-1 was synthesized using a solvothermal method in a one-step procedure. In brief, 2.20 mmol of Cu(NO_3_)_2_·3H_2_O was dissolved in 7 mL of deionized water. Separately, 1.20 mmol of H_3_BTC (benzene-1,3,5-tricarboxylic acid) was dissolved in 7 mL of ethanol. The two solutions were then mixed and sonicated for 20 minutes to form a homogeneous solution. This mixture was transferred into a Teflon-lined stainless-steel autoclave and heated at 120 °C for 24 hours. After cooling to room temperature, the turquoise blue precipitate was collected by centrifugation. The precipitate was washed several times with deionized water and ethanol, then dried for 12 hours at 80 °C in a vacuum oven.^43^

#### 2.3.2. Preparation of NiFe_2_O_4_

NiFe_2_O_4_, the core section of the core–shell structure, was synthesized using a solvothermal method. Initially, 1.8 g of FeCl_3_, 0.71 g of NiCl_2_, and 5.75 g of sodium acetate were dissolved in 50 mL of ethylene glycol using an ultrasonic probe to ensure complete dissolution. The resulting solution was then transferred into a Teflon-lined stainless-steel autoclave and heated for 8 hours at 200 °C. After cooling to room temperature, a brown precipitate was obtained. This precipitate was washed several times with distilled water and ethanol, followed by drying under vacuum for 8 hours at 60 °C. The brown powder, exhibiting magnetic properties, was then ready for further steps.

#### 2.3.3. Preparation of NiFe_2_O_4_@HKUST-1

The NiFe_2_O_4_@HKUST-1 core–shell nanocomposite was synthesized by coating NiFe_2_O_4_ with HKUST-1 using a layer-by-layer assembly method. To enhance the surface functionality, 0.35 g of NiFe_2_O_4_ was first dispersed in 10 mL of ethanol containing 0.06 mL of mercaptoacetic acid and stirred for 24 hours. The modified NiFe_2_O_4_ was then collected using an external magnetic field and washed repeatedly with distilled water and ethanol. Subsequently, 0.35 g of the modified NiFe_2_O_4_ was dispersed in 5 mL of ethanol containing 5 mg of Cu(NO_3_)_2_·3H_2_O. The mixture was briefly heated to 50 °C and stirred for 30 minutes. After magnetic separation, the solid was transferred into a solution of 53 mg of benzene-1,3,5-tricarboxylic acid (BTC) in 5 mL of ethanol, heated to 50 °C, and stirred for 60 minutes. The resulting product was again magnetically separated, thoroughly rinsed with ethanol, and dried at 50 °C for 30 minutes. This process constituted one complete coating cycle, and it was repeated 10 times to successfully form the NiFe_2_O_4_@HKUST-1 core–shell structure.

#### 2.3.4. Preparation of NiFe_2_O_4_@HKUST-1/GO

**Scheme 1:**
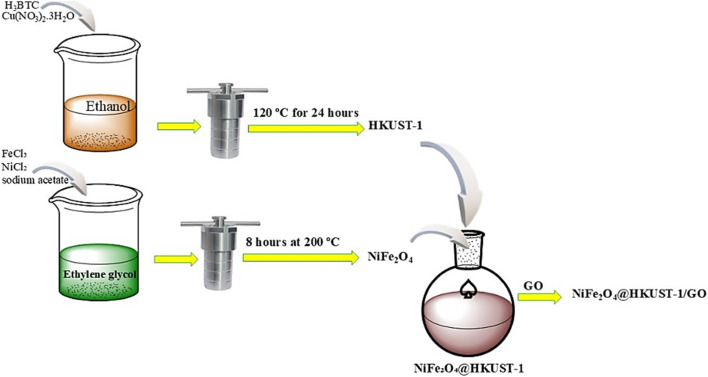
The synthesis procedure of NiFe_2_O_4_@HKUST-1/GO composite.

The synthesis of NiFe_2_O_4_@HKUST-1/GO began by dispersing GO powder into the H_3_BTC solution. During the final cycle of the procedure described in Section 2.3.3, GO was added to the ethanol solution of BTC at a ratio of 10% w/w relative to the total mass of the final composite. To ensure a homogeneous mixture, the solution was subjected to ultrasonic treatment before the heating step. This step facilitated the uniform distribution of GO within the NiFe_2_O_4_@HKUST-1 structure, resulting in the desired NiFe_2_O_4_@HKUST-1/GO composite, as shown all procedures in Scheme 1.

### 2.4. Adsorption experiment

First, 0.05 g of the adsorbent was added to 50 mL of aqueous Congo Red solution (5 ppm). The mixture was shaken at room temperature (300 rpm) for varying time intervals (0–60 min). At different times (5, 10, 15, 20, 30, 40, and 60 min), 5 mL of the mixture was taken, and the precipitate was separated using a magnet. The samples were collected and analyzed for the residual Congo Red concentration using a UV-Vis spectrophotometer in the wavelength range of 200–800 nm. The maximum adsorption capacity was determined based on the changes in Congo Red concentration over time.

### 2.5. Simulation studies

**Fig. 1:**
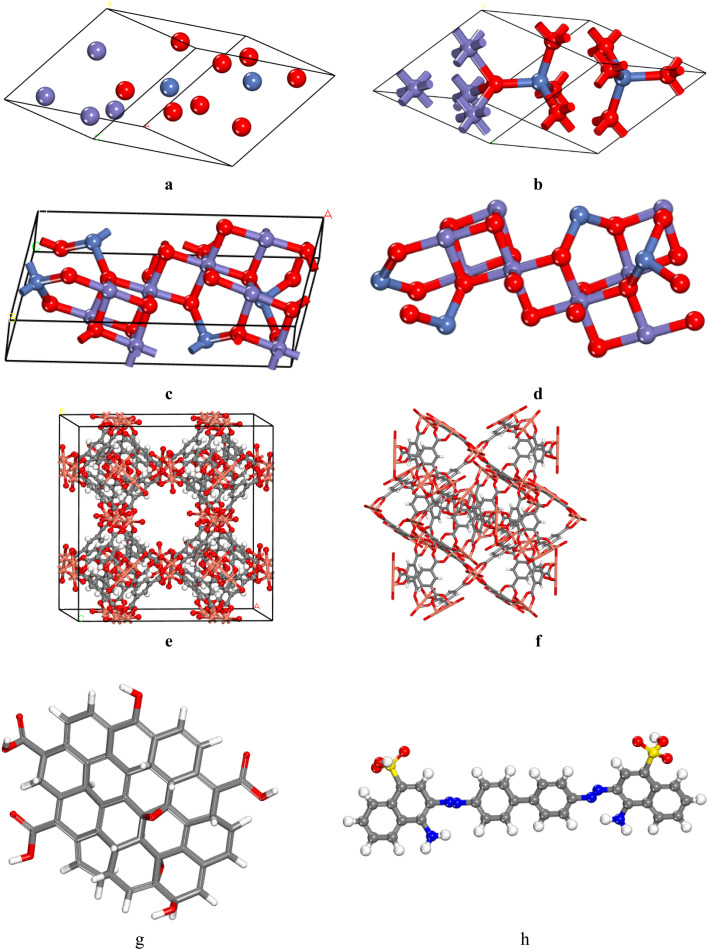
(a and b) The crystal structures of ball and stick of Fe_2_NiO_4_. (c) The crystal structure of Fe_2_NiO_4_ (311), (d) Fe_2_NiO_4_ (311) without crystal structure, (e); the crystal structure of HKUST-1, (f) HKUST-1 without crystal structure, (g) GO structure and (h) Congo Red. O atoms in red, N atoms in blue, Cu atoms in orange, Ni atoms in violet and C atoms in gray.

Fe_2_NiO_4_, exhibiting a cubic structure (Code: mp-22684, Point Group: *Fd*3̄*m*) with lattice parameters *a* = *b* = *c* = 5.996 Å and *α* = *β* = *γ* = 60.000°, was obtained from the Materials Project database at https://legacy.materialsproject.org/materials/mp-24941/# (Fig. 1a and b). The crystal was grown on the (*hkl*: 311) plane with a thickness of 13.997 Å to form Fe_2_NiO_4_ (311). The vacuum thickness was set to 0.00, and the slab position was maintained at 0.0 (Fig. 1c), then to construct composite, without cell is needed (Fig. 1d). HKUST-1 was download from ChemTube3D (Fig. 1e and f). The optimized graphene oxide (GO), containing epoxy, hydroxyl, and carboxyl functional groups, and Congo Red was designed using the software (Fig. 1g).

Monte Carlo (MC) simulations are powerful statistical methods used to explore and analyze systems by sampling configurations based on probabilistic rules. In your context, Monte Carlo simulations were employed to investigate the total energies and mechanisms underlying complex-carrier interactions within a material system, using the Materials Studio (MS) software package.^3,44–46^ The calculations involved 10 cycles with a group-based cutoff radius of 12.5 Å, emphasizing van der Waals and electrostatic interactions to accurately model the system's behavior.^47^ The Universal Force Field (UFF) along with assigned atomic charges was employed to accurately predict the most favorable adsorption sites within the nanocomposite structure. This approach enabled detailed modeling of intermolecular interactions, particularly within an adsorption distance of less than 10 Å, which is critical for simulating realistic dye–adsorbent interactions. The UFF's ability to handle diverse atom types-including transition metals and organic ligands-makes it well-suited for studying hybrid systems like NiFe_2_O_4_@HKUST-1/GO. By incorporating electrostatic and van der Waals contributions, the simulation effectively captured key aspects of the adsorption mechanism and helped correlate structural features with adsorption performance.^48^ In this Monte Carlo simulation, the calculations for catalyst formation were conducted in the presence of 10 water molecules. The purpose of including water molecules in the simulation could be to account for the influence of the solvent (water) on the catalyst's behavior and properties. During the simulation procedure, the atom-based method was employed to precisely calculate the potential energy of the complex, enabling a thorough assessment of the system's interactions.

## 3. Results and discussion

### 3.1. Characterization results

**Fig. 2:**
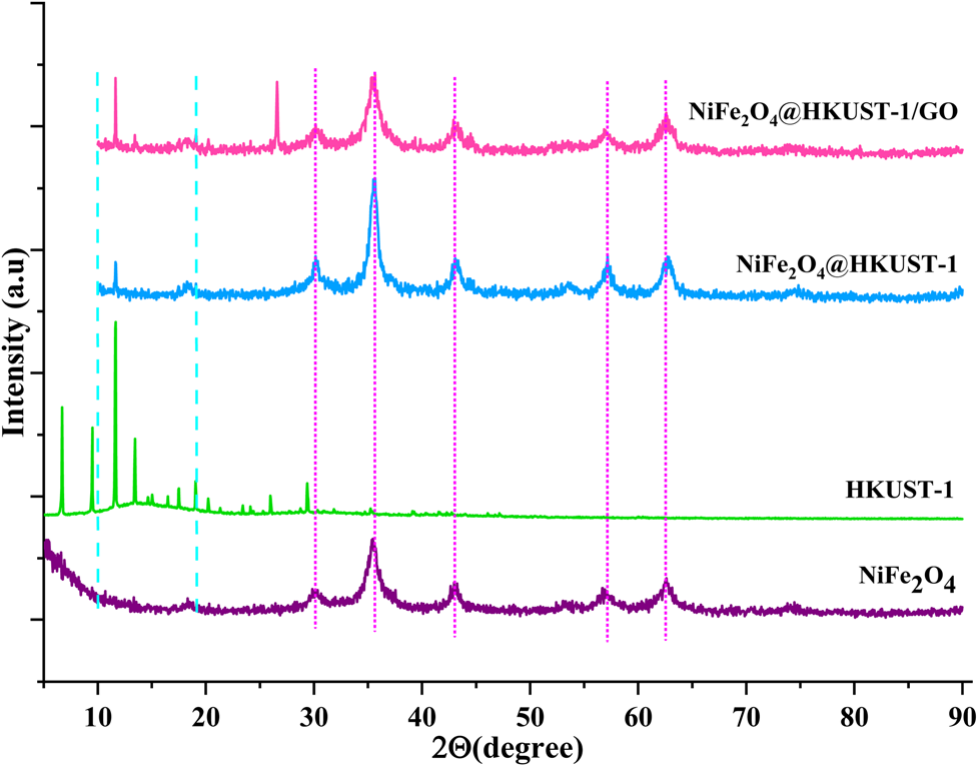
XRD patterns of the samples.

The X-ray diffraction (XRD) patterns of HKUST-1, NiFe_2_O_4_, NiFe_2_O_4_@HKUST-1, and NiFe_2_O_4_@HKUST-1/GO are shown in Fig. 2. For HKUST-1, characteristic diffraction peaks were observed in the 2*θ* range of 10–15°, corresponding to its MOF structure. NiFe_2_O_4_ exhibited distinct peaks at 2*θ* values of 19°, 30°, 35°, 42°, 54°, 59°, 61°, and 74°, consistent with standard data from the Joint Committee on Powder Diffraction Standards (JCPDS). In the XRD pattern of NiFe_2_O_4_@HKUST-1, diffraction peaks corresponding to both NiFe_2_O_4_ and HKUST-1 were present, confirming the successful formation of the NiFe_2_O_4_@HKUST-1 nanocomposite. Additionally, in the pattern of NiFe_2_O_4_@HKUST-1/GO, the characteristic GO peak around 10° was also observed, indicating the incorporation of GO into the composite,^49^ it is hard to observe it in NiFe_2_O_4_@HKUST-1, so that the diffraction pattern of NiFe_2_O_4_@HKUST-1/GO is similar to NiFe_2_O_4_@HKUST-1.

**Fig. 3:**
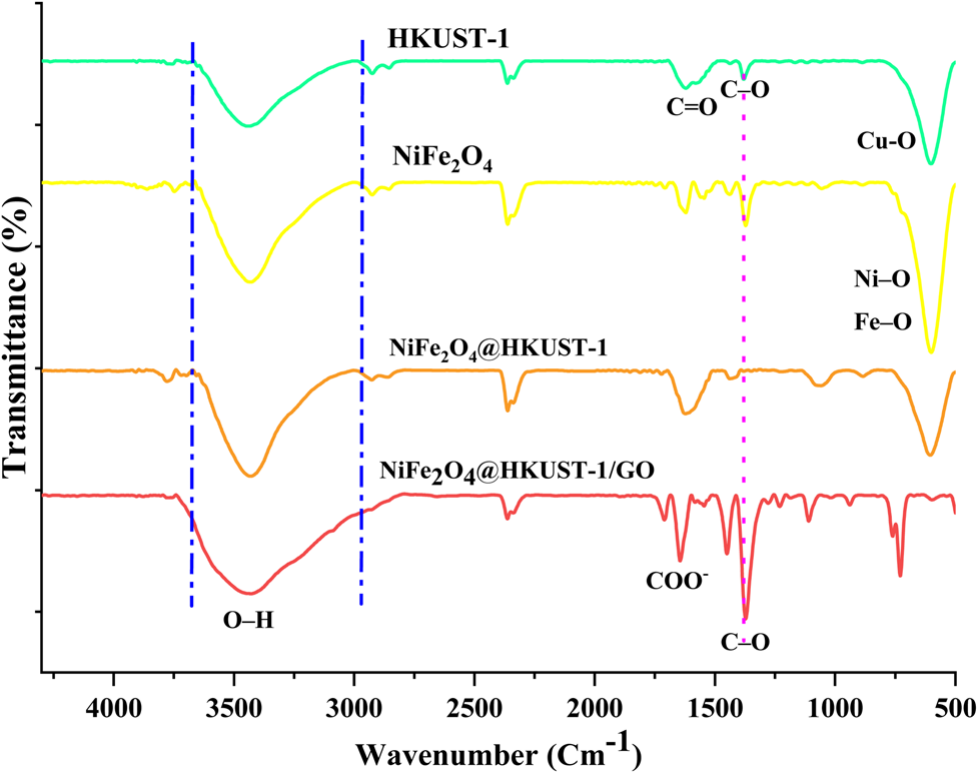
The FTIR spectra of the samples.

The FT-IR spectra of NiFe_2_O_4_, HKUST-1, NiFe2O4@HKUST-1, and NiFe_2_O_4_@HKUST-1/GO are presented in Fig. 3. The band observed at 1374 cm^−1^ is attributed to the C–O bond of BTC, while the bands at 1452 cm^−1^ and 1560 cm^−1^ correspond to the C=O bond of BTC. The band at 1645 cm^−1^ is associated with the C=C stretch in the aromatic ring, and the band at 1717 cm^−1^ is attributed to the COO^−^ group of BTC. The bands in the range of 1310–500 cm^−1^ are ascribed to the out-of-plane vibrations of BTC. Additionally, the band between 550–530 cm^−1^ corresponds to the intrinsic stretching vibration of metal bonds at the tetrahedral site (Ni–O and Fe–O bonds).^50,51^ The bands at 488, 671 and 810 cm^−1^ were assigned to deformation vibration of Fe–OH bonds. The bands at 3800–3400 cm^−1^ and 1600 cm^−1^ could be ascribed to O–H stretching vibration of H_2_O absorbed by sample and O–H bonds of the surface.^52^ The peak of GO was at 4000–2700 cm^−1^ that was merged with peak of O–H stretching band.

**Fig. 4:**
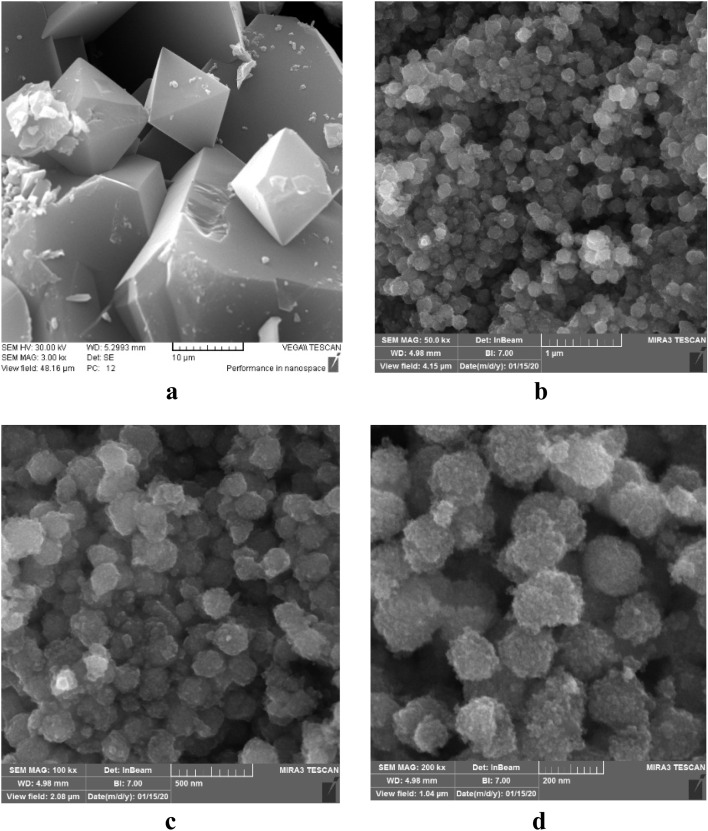
SEM images of (a) HKUST-1, FESEM images of (b) NiFe_2_O_4_, (c) NiFe_2_O_4_@HKUST-1 and (d) NiFe_2_O_4_@HKUST-1/GO.

The morphology of the nanocomposite is illustrated in the SEM images shown in Fig. 4. As depicted in Fig. 4a, HKUST-1 exhibits an octahedral shape with average diameters ranging from 10 to 20 μm. The synthesis process at the applied temperature resulted in the formation of cubic crystals with well-defined sharp edges. Fig. 4(b–d) reveal that NiFe_2_O_4_, NiFe_2_O_4_@HKUST-1, and NiFe_2_O_4_@HKUST-1/GO all display spherical morphologies. NiFe_2_O_4_, in particular, exhibits a microsphere structure with varying sizes, suggesting that NiFe_2_O_4_ serves as the core, encapsulated by the HKUST-1 shell.

**Fig. 5:**
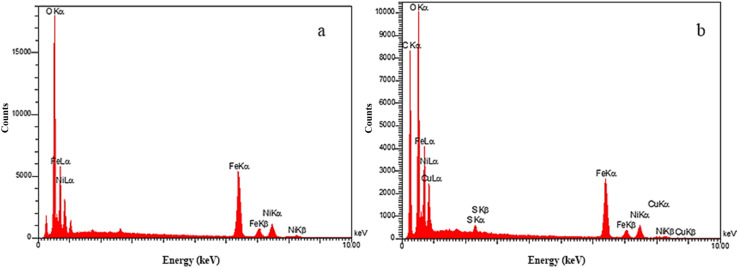
EDS spectra of (a) NiFe_2_O_4_ and (b) NiFe_2_O_4_@HKUST-1/GO.

The EDS spectrum of NiFe_2_O_4_ (Fig. 5a) displays characteristic peaks at 0.5, 0.7, 0.8, 6.5, 7.1, 7.5, and 8.2 keV, corresponding to O Kα, Fe Lα, Ni Lα, Fe Kα, Fe Kβ, Ni Kα, and Ni Kβ, respectively, confirming the presence of the core elements. In the spectrum of NiFe_2_O_4_@HKUST-1/GO (Fig. 5b), additional peaks appear at 0.2, 0.5, 0.9, 8.1, and 8.9 keV, which can be attributed to C Kα, O Kα, Cu Lα, Cu Kα, and Cu Kβ, respectively-consistent with the incorporation of HKUST-1 as the shell material (as shown Tables 1S and 2S†). The remaining peaks align with those observed for NiFe_2_O_4_, verifying the successful formation of the core–shell composite structure.

**Fig. 6:**
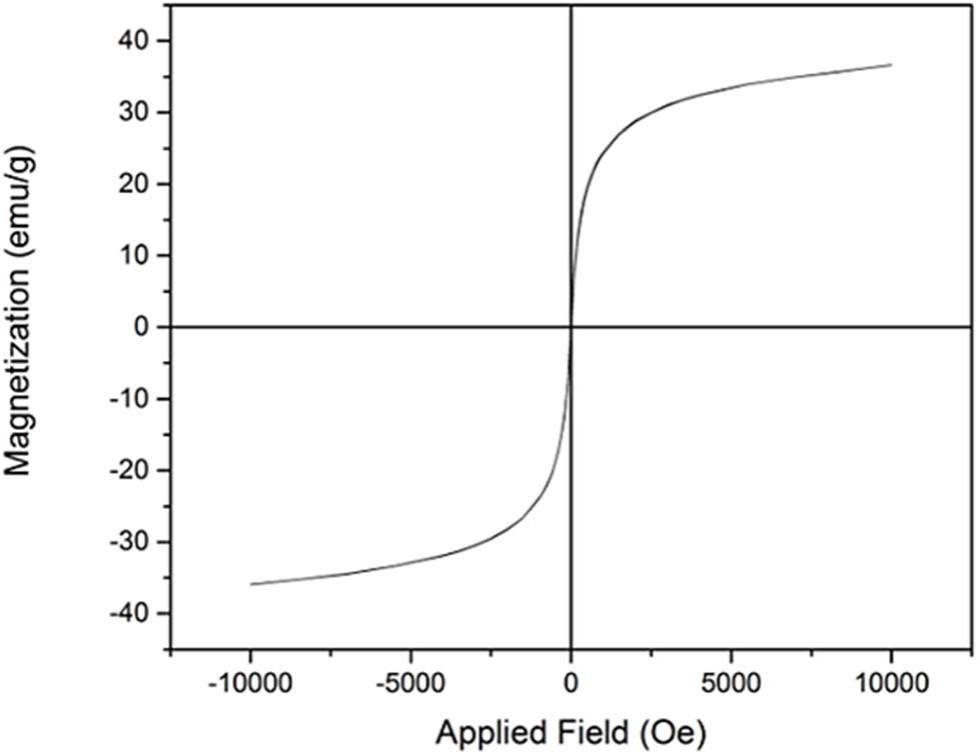
Magnetic properties of NiFe_2_O_4_@HKUST-1/GO composite using a vibrating sample magnetometer (VSM).

The magnetic properties of the composite were evaluated using a vibrating sample magnetometer (VSM). As shown in Fig. 6, the magnetic hysteresis curve recorded at room temperature exhibits a saturation magnetization (*M*_s_) of 35 emu g^−1^, a remanent magnetization (*M*_r_) of approximately 6 emu g^−1^, and a coercivity (*H*_c_) close to 0 emu g^−1^. These results indicate the composite exhibits soft magnetic behavior.

**Fig. 7:**
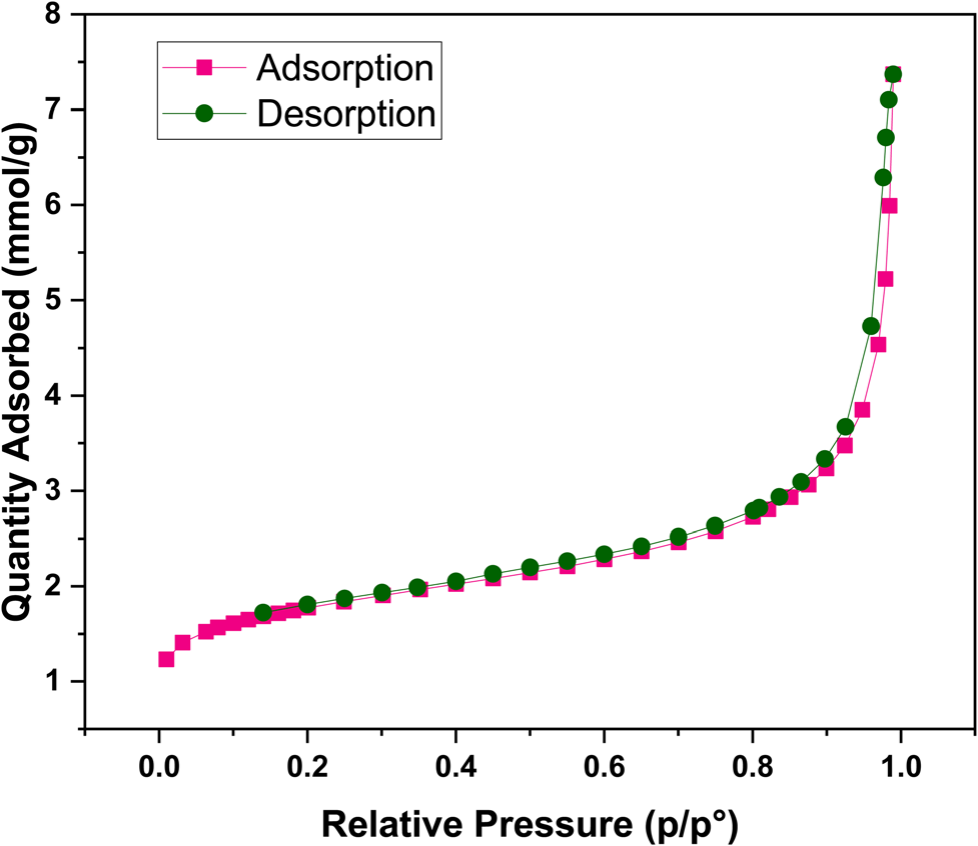
N_2_ adsorption–desorption isotherms of NiFe_2_O_4_@HKUST-1/GO.

Based on the IUPAC classification, the NiFe_2_O_4_@HKUST-1/GO nanocomposite exhibits a type IV nitrogen adsorption–desorption isotherm (shown in Fig. 7), indicative of a mesoporous structure. BET analysis revealed a specific surface area of 138.46 m^2^ g^−1^, a total pore volume of 0.02623 cm^3^ g^−1^, and an average pore diameter of 45.44 Å. These findings confirm the presence of well-developed mesopores, which facilitate enhanced surface interactions and adsorption capacity. Such features are particularly advantageous for applications like dye removal from aqueous solutions, where multilayer adsorption plays a key role.

### 3.2. Adsorption of Congo Red

#### 3.2.1. Effect of pH on Congo Red adsorption

**Fig. 8:**
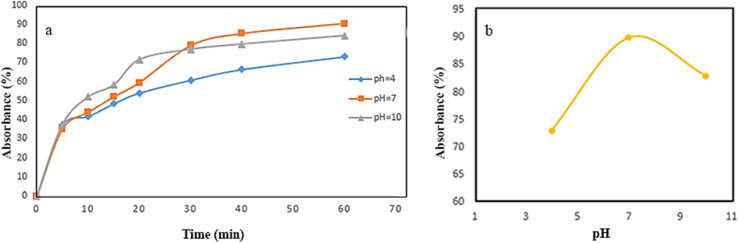
(a) Absorbance percentage *versus* time for three different pHs of absorbent solution, (b) absorbance percentage *versus* pH on NiFe_2_O_4_@HKUST-1/GO.

pH is one of the most critical factors influencing dye removal from aqueous solutions. In this study, the effect of pH on the adsorption of Congo Red was evaluated at pH values of 4, 7, and 10. As shown in Fig. 8, dye removal efficiency increased with pH, reaching a maximum at pH 7. However, a significant decrease in adsorption was observed at pH 10, indicating that neutral conditions are most favorable for Congo Red removal using the synthesized composite.

Therefore, two sorption mechanisms can be considered: chemisorption and physical adsorption. This trend can be attributed to the nature of surface charge interactions and the dominant adsorption mechanisms at different pH levels. At pH 4, the sorbent surface is predominantly protonated, facilitating strong electrostatic attraction between the positively charged surface and the negatively charged anionic Congo Red dye. As the pH approaches neutrality (pH 7), optimal adsorption occurs, likely due to a balanced interplay between surface charge, dye ionization state, and available binding sites. Beyond this point, at pH 10, the sorbent surface becomes increasingly deprotonated and negatively charged, leading to significant electrostatic repulsion between the surface and the dye anions. This repulsion weakens the interaction, thus reducing dye uptake. Furthermore, post-adsorption characterization data presented in Fig. 13. Confirm that chemisorption was the dominant mechanism, as evidenced by shifts in functional group signals and changes in crystallinity—indicating the formation of specific chemical bonds between the sorbent and dye molecules.

#### 3.2.2. Amount of adsorbent

**Fig. 9:**
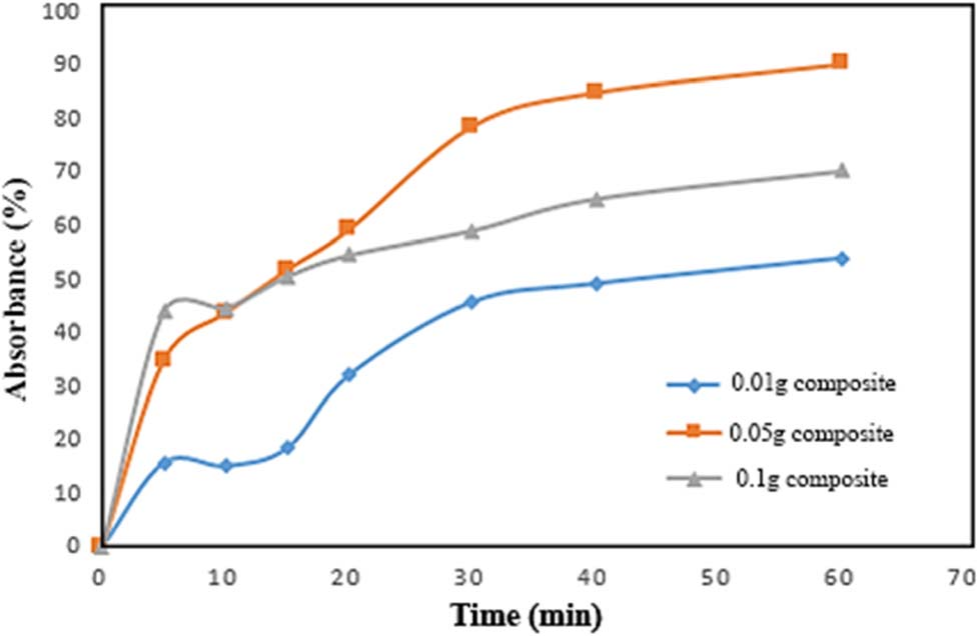
Absorbance percentage *versus* time for three different weights of composite.

The influence of adsorbent dosage on Congo Red removal was investigated at pH 7 and room temperature using 0.01, 0.05, and 0.1 g of the composite material. As shown in Fig. 9, the optimal adsorption was achieved with 0.05 g of adsorbent. At a lower dosage (0.01 g), fewer active sites were available, resulting in reduced dye uptake. Interestingly, increasing the dosage to 0.1 g led to a decrease in adsorption efficiency, likely due to the aggregation of excess adsorbent particles, which reduces the effective surface area and leaves some active sites unutilized.

#### 3.2.3. Effect of contact time

**Fig. 10:**
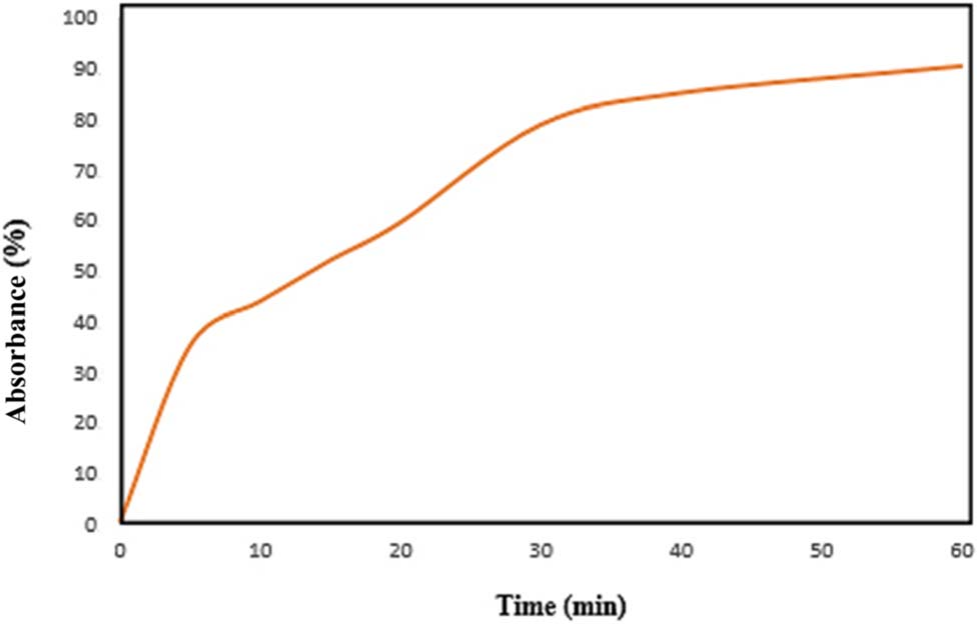
Effect of contact time on absorbance percentage.

To assess the effect of contact time on adsorption efficiency, experiments were conducted over a 60-minute period. As shown in Fig. 10, dye removal increased with time, reaching approximately 90% removal within 60 minutes. The adsorption process can be divided into three distinct phases: (i) a rapid uptake during the initial 0–7 minutes, characterized by a steep slope due to the abundance of available active sites; (ii) a slower, more gradual uptake from 7 to 35 minutes as active sites begin to fill; and (iii) a plateau phase from 35 to 60 minutes, where the adsorption rate slows significantly, indicating that most active sites are occupied and equilibrium is approaching.

#### 3.2.4. Effect of dye concentration

**Fig. 11:**
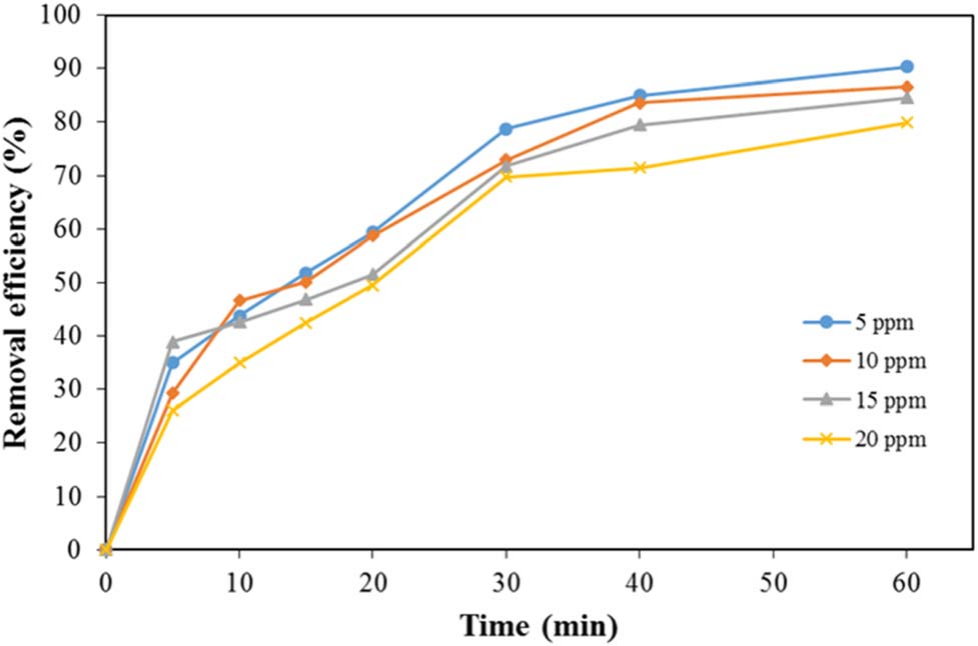
Removal efficiency of the composite affected by dye concentration.

To evaluate the effect of initial dye concentration on adsorption performance, Congo Red solutions with concentrations of 5, 10, 15, and 20 ppm were prepared and tested. As illustrated in Fig. 11, dye adsorption increased with time across all concentrations. The initial adsorption rate was rapid, particularly within the first 40 minutes, due to the abundance of available active sites. After approximately 60 minutes, the system approached equilibrium, and the adsorption rate slowed, as reflected in the reduced slope between 40–60 minutes. The adsorption efficiencies corresponding to each concentration are presented in Table 1.

**Table 1:** Effects of dye concentration on adsorbing dye

Concentration (ppm)	20	15	10	5
Removal efficiency (%)	80	84.6	86.7	90.3

### 3.3. Kinetics for the adsorption of Congo Red

To gain more general data about the adsorption kinetic of this process, some kinetic models such as pseudo-first-order and pseudo-second-order were applied to the experimental data at pH ∼ 7. Pseudo-first-order equation is based on the relation of the adsorption uptake quantity with time^53^ and it is given in eqn (1).

1 ln(*q*_e_ − *q*_*t*_) = ln *q*_e_ + *k*_1_*t*

where *k*_1_ is the pseudo-first-order rate constant (min^−1^), *q*_*t*_ is the amount of dye adsorbed at time *t* (mg g^−1^), and *q*_e_ is the amount of dye adsorbed at equilibrium (mg g^−1^) and pseudo-second-order model is based on the supposition of chemisorption of the dye on the sorbent. It is expressed as eqn (2).2
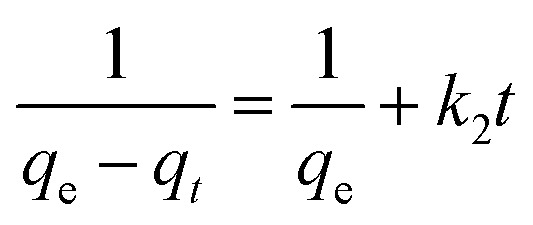
where *q*_e_ is the amounts of congo Red adsorbed (mg g^−1^) at equilibrium in pseudo-second-order model and *k*_2_ is the pseudo-second-order rate constant (g mg^−1^ min).

**Fig. 12:**
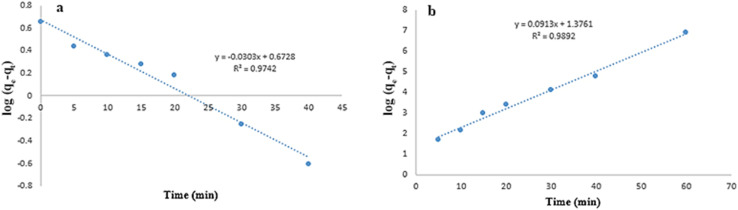
Adsorption kinetics for (a) pseudo-first-order, (b) pseudo-second-order models.

As it can be seen in Fig. 12 and Table 2, the calculated linear regression correlation coefficient (*R*^2^) of pseudo-second-order suggested that the adsorption of dye process on NiFe_2_O_4_@HKUST-1/GO follows the pseudo-second-order model. Additionally, the *χ*^2^ parameter has been calculated for both pseudo firs order and pseudo second order models, as it illustrated in Table 2. The *χ*^2^ parameter values validates that the pseudo-second-order kinetic is the determinative process kinetics for dye adsorption of NiFe_2_O_4_@HKUST-1/GO.

**Table 2:** Kinetic parameter

Reaction	*R* ^2^	*χ* ^2^
Pseudo-first-order	0.9742	1.1341
Pseudo-second-order	0.9826	0.3487

**Fig. 13:**
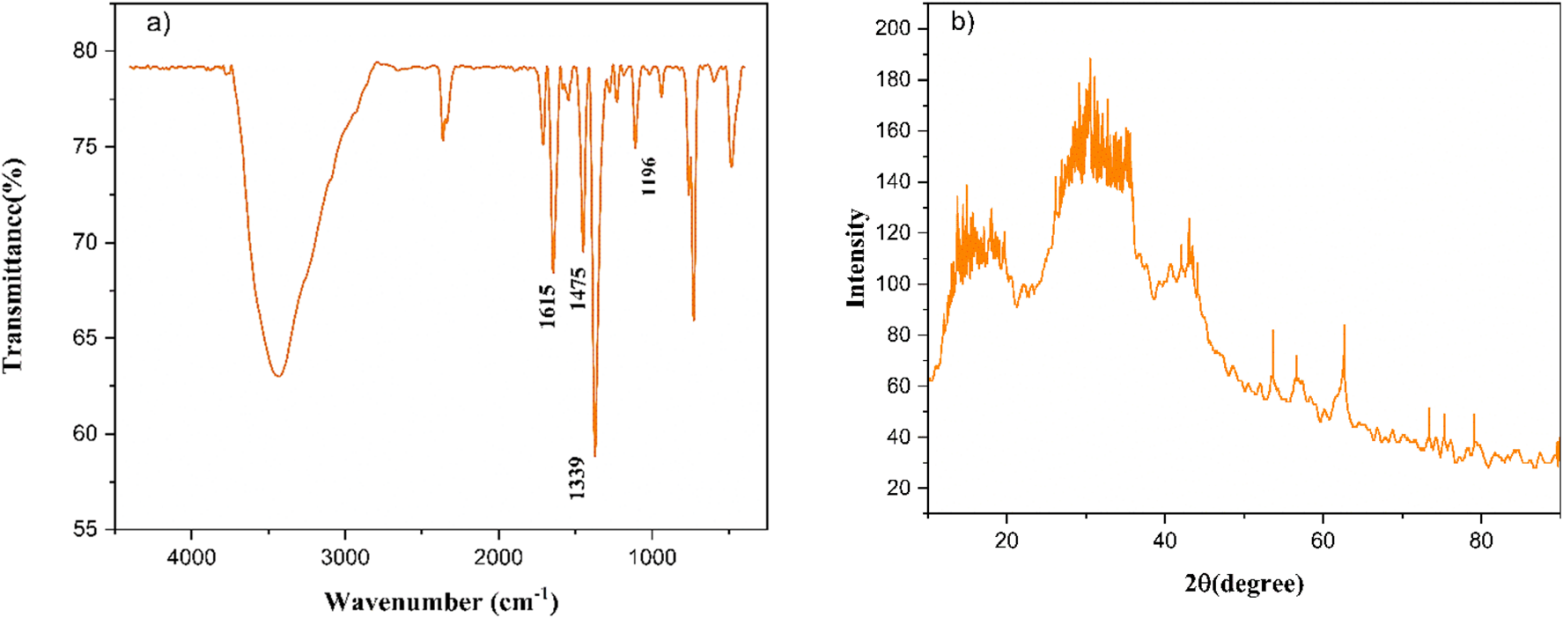
After adsorption analysis: (a) FT-IR spectrum and (b) XRD pattern for NiFe_2_O_4_@HKUST-1/GO after adsorption of Congo Red.

Moreover, the IR and XRD data after adsorption are presented in Fig. 13. The IR spectrum clearly indicates the presence of functional groups associated with Congo Red. The peaks observed at 1196, 1336, 1531, and 1612 cm^−1^ correspond to –SO_3_H, –NO_2_, and characteristic diazo groups of red azo dyes. In the XRD pattern, the appearance of an amorphous phase is evident. Moreover, noticeable peak shifts and broadening are observed, indicating a reduction in crystallinity and confirming the chemical interaction between Congo Red and the adsorbent.

### 3.4. Adsorption isotherms

**Fig. 14:**
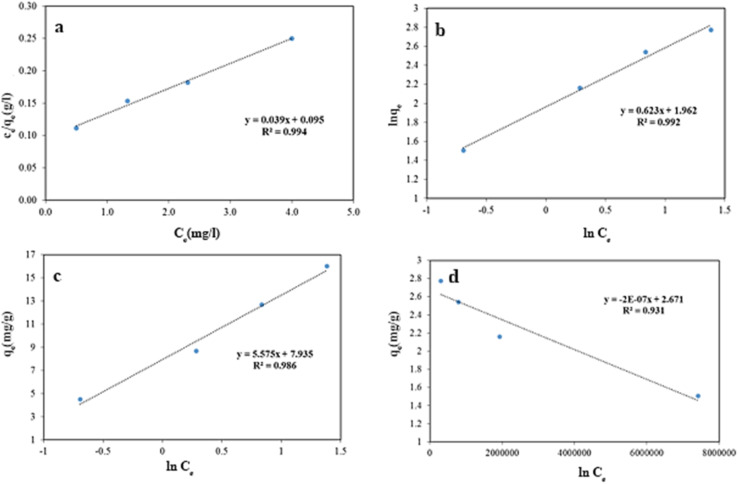
(a) Langmuir, (b) Freundlich (c) Temkin (d) Dubinin–Radushkevich isotherms models.

To describe the adsorption isotherms, we checked out Langmuir isotherm, Freundlich isotherm, Temkin model and Dubinin–Radushkevich (D–R) model. In Fig. 14, all of the isotherm models are shown. Based on Langmuir model, the adsorption equation is expressed as eqn (3).3
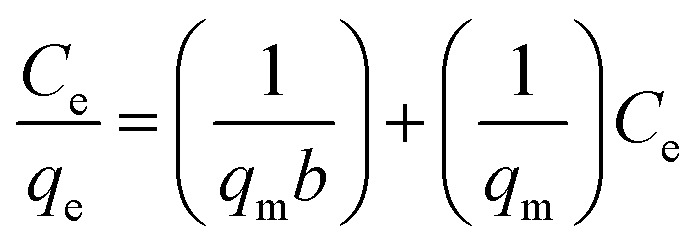
where *q*_e_ (mg g^−1^) is the amount of the dye adsorbed at equilibrium, *C*_e_ is the equilibrium concentration of the dye in solutions (mg L^−1^), *q*_m_ (mg g^−1^) is the Langmuir constant demonstrating maximum capacity, and *b* is the Langmuir constant related to the sorption energy.

The Freundlich isotherm is widely considered an empirical model without a defined physical basis. It is typically used to describe multilayer adsorption on heterogeneous surfaces, where the adsorbent possesses a range of sites with varying affinities for the adsorbate. This model is especially suitable for systems where surface uniformity cannot be assumed.^54^ Its equation is expressed as eqn (4).4
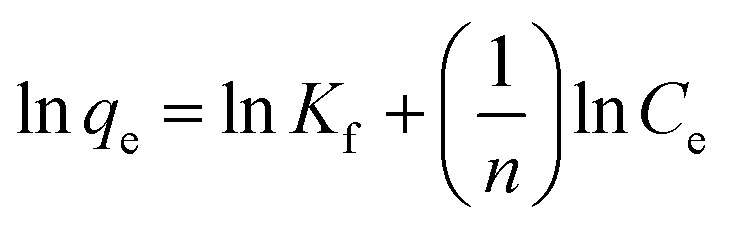
where *K*_f_ and *n* are Freundlich isotherm constants which are related to absorption capacity and intensity, respectively.

The Temkin model, is for multi-layer adsorption process. Very high and low concentration amount of adsorbent are refused. The Temkin model is presented in eqn (5).5
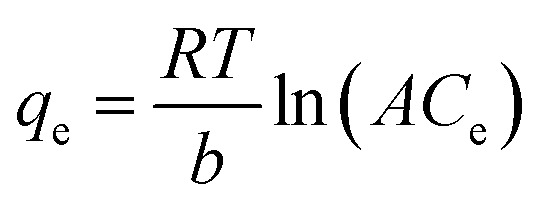
where *A* (L g^−1^) and *b* (J mol^−1^) are Temkin isotherm constants.

The Dubinin–Radushkevich model was suggested as an experimental isotherm to represent the adsorption of vapors on solids.^55^ The nonlinear D–R model is presented in eqn (6) and (7).

6 *q*_e_ = *q*_mD–R_ e^−*K*_DR_^ *ε*^2^

7
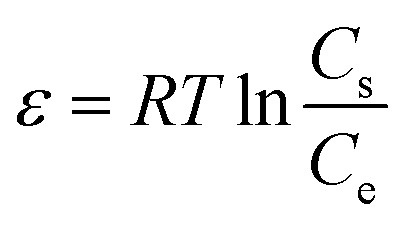
where *q*_mD−R_ (mg g^−1^) is the maximum adsorbed value, *K*_DR_ (mol^2^ kJ^−2^) is the constant of model, *ε* (kJ mol^−1^) is the adsorption potential and *C*_s_ (mg L^−1^) is the solubility of adsorbents.

As can be concluded from Fig. 14 and Table 3, adsorption process is followed by Langmuir and Freundlich isotherm models, because the higher value of regression (*R*^2^) coefficient indicates a good agreement between the parameters and the best fitting of these models. Moreover, the *χ*^2^ parameter values have been presented in Table 3. As the same, and these values further confirm that the Langmuir and Freundlich isotherm models exhibit superior correlation and alignment with the experimental adsorption data, demonstrating their greater applicability in describing the system behavior.

**Table 3:** Regression coefficients of the four models

Adsorption isotherm	Langmuir	Freundlich	Temkin	D–R
*R* ^2^	0.994	0.992	0.986	0.931
*χ* ^2^	0.192	0.216	0.342	1.328

### 3.5. Effect of recycled NiFe_2_O_4_@HKUST-1/GO on Congo Red adsorption

**Fig. 15:**
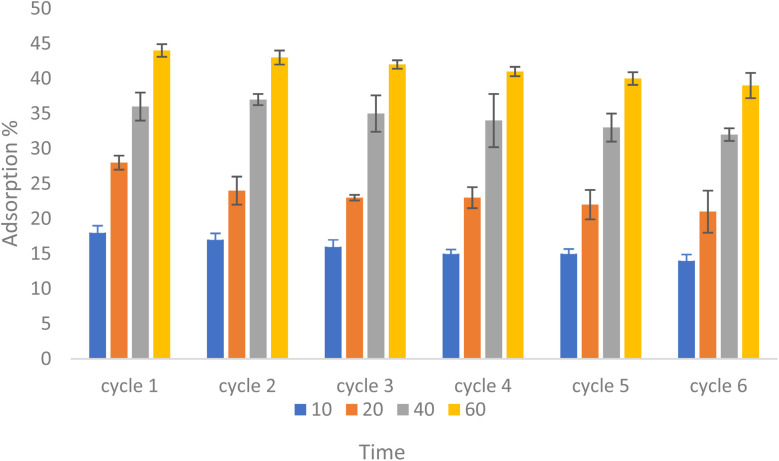
Cycles of reusability of NiFe_2_O_4_@HKUST-1/GO.

To study the feasibility of reusability of NiFe_2_O_4_@HKUST-1/GO as an adsorbent, it was washed and the number of efficiency cycles were determined by successive repeating adsorption and desorption cycles. For this purpose, 0.05 g of the adsorbent was added to a 50 mL solution with a dye concentration of 5 ppm at pH ∼7, and after 60 minutes, all of the adsorbent was separated from the solution and rinsed with water and distilled ethanol several times to complete the desorption process. After desorption and drying, all the adsorbent powder was added to 5 mL of 5 ppm solution and the cycle was repeated. As shown in Fig. 15, after four cycles and due to the lack of significant change in the percentage of the pollutant absorption in each cycle, it can be concluded that the reusability of this nanocomposite is economical and effectual.

### 3.6. Comparing the adsorption of CR by NiFe_2_O_4_@HKUST-1/GO and its constituents

The adsorption of HKUST-1, HKUST-1/GO, NiFe_2_O_4_@HKUST-1 as the constituents was investigated and compared with NiFe_2_O_4_@HKUST-1/GO as the whole composite. To conduct the adsorption experiment, 50 mL of a 5 ppm Congo Red (CR) solution was prepared and distributed into five separate containers. In each container, 0.05 g of the respective adsorbent (HKUST-1, HKUST-1/GO, NiFe_2_O_4_@HKUST-1, or NiFe_2_O_4_@HKUST-1/GO) was added. The mixtures were stirred for 1 hour to ensure proper contact between the adsorbent and CR solution. Samples were taken at 7 different time intervals: 5, 10, 15, 20, 30, 40, and 60 minutes. For the solutions containing HKUST-1 and HKUST-1/GO, the CR and adsorbent were separated using centrifugation. In contrast, for the solutions with NiFe_2_O_4_@HKUST-1 and NiFe_2_O_4_@HKUST-1/GO, separation was achieved simply by applying an external magnet due to the magnetic properties of these adsorbents.

**Fig. 16:**
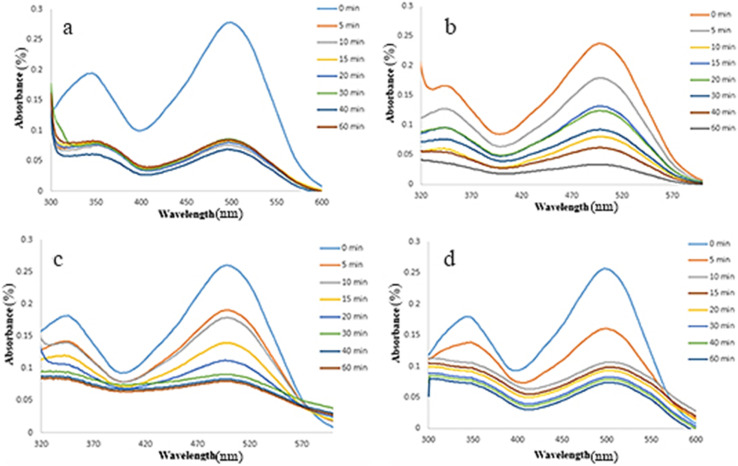
Adsorption percentage of CR *versus* wavelength in different times by the applied adsorbents; (a) HKUST-1, (b) HKUST-1/GO, (c) NiFe_2_O_4_@HKUST-1, and (d) NiFe_2_O_4_@HKUST-1/GO.

**Fig. 17:**
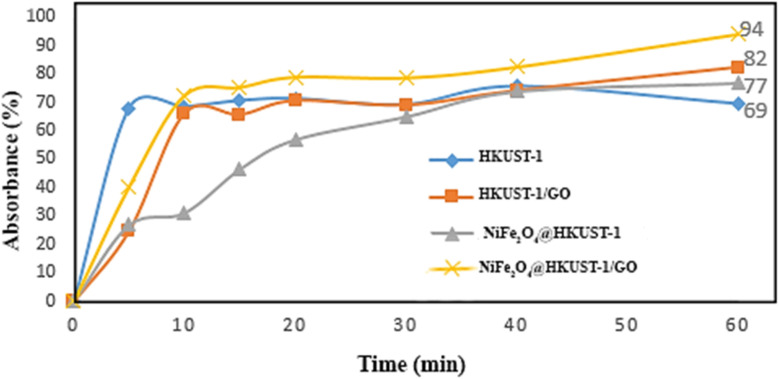
Adsorption percentage of CR during 60 min for the applied adsorbents; HKUST-1, HKUST-1/GO, NiFe_2_O_4_@HKUST-1, and NiFe_2_O_4_@HKUST-1/GO.

Finally, UV-Vis spectrophotometer was used to determine the concentration of remaining dye, whose result is illustrated in Fig. 16(a–d). The changes of adsorption *versus* time are illustrated in Fig. 17, in which a comparison between the pollutant dye adsorption by the prepared three-component adsorbent (∼94%) and some of its components (69–82%) shows that we can consider NiFe_2_O_4_@HKUST-1/GO as a more efficient adsorbent than its components, individually.

### 3.7. Comparison the removal of Congo Red with other composite materials

To highlight the effectiveness of the as-synthesized composite material for Congo Red (CR) removal, a comparative analysis with other reported composite adsorbents is provided. The comparison includes parameters such as maximum adsorption capacity, operating pH, and equilibrium time. As shown in the table below, the as-synthesized composite demonstrates competitive or superior performance in terms of adsorption capacity and removal efficiency. This suggests that the introduced material is a promising candidate for the effective removal of CR from aqueous environments, offering advantages such as high adsorption potential, suitable operational pH, and reasonable kinetics compared to existing materials (Table 4).

**Table 4:** Comparison of adsorption capacity (mg g^−1^) adsorption capacity and equilibrium time in pH for the removal of CR dye

Adsorbent	Strategy	Adsorption capacity	Removal efficiency (%)	Contact time (min)	pH	Ref.
BiO–Ag(0)/C_3_N_4_@ZIF-67	Photodegradation	1000 mg g^−1^	90%	120	—	56
MOF-Fe	Adsorption	775.19 mg g^−1^	—	12 h	7.6	57
MOF-Co		628.93 mg g^−1^				
ZnAl/SP	Adsorption	185.185 mg g^−1^	—	2.5 h	5, 6, and 7	58
ZnAl/EC		144.928 mg g^−1^				
MnFe_2_O_4_-GO	Adsorption	9.89 mg g^−1^	—	60	—	59
Mg–Al LDH and MoS_2_/Mg–Al LDH	Adsorption	56.2 and 116.41 mg g^−1^	—	45 and 80	3	60
Spathodea campanulata flowers (SCAC)	Adsorption	59.27 mg g^−1^	—	180	7	61
NiFe_2_O_4_@HKUST-1/GO	Adsorption	25.64 mg g^−1^	—	60	7	—

### 3.8. Monte Carlo simulations

#### 3.8.1. The adsorption of NiFe_2_O_4_ and HKUST-1 surface

**Fig. 18:**
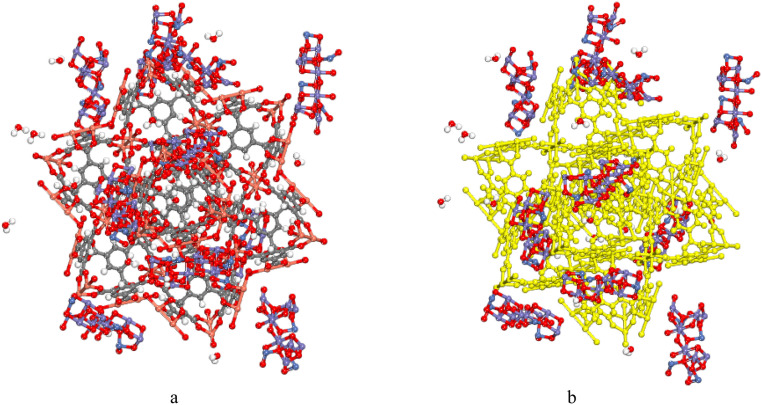
(a and b) The adsorption of NiFe_2_O_4_ (311) on HKUST-1 surface to form NiFe_2_O_4_@HKUST-1 by Monte Carlo simulation.

Monte Carlo simulations, integrated with a simulated annealing approach, were employed to investigate the orientation, adsorption behavior, and interfacial interactions between the NiFe_2_O_4_ surface and HKUST-1, aiming to construct the NiFe_2_O_4_(311)@HKUST-1 interface,^62^ which each simulation comprised three heating cycles, with 15 000 steps per cycle. Monte Carlo (MC) simulations, a widely utilized stochastic method in computational chemistry, enabled the exploration of the adsorption behavior of NiFe_2_O_4_ (311) on the HKUST-1 surface in a realistic and dynamic context. The adsorption of inorganic NiFe_2_O_4_ (311) compounds onto the HKUST-1 surface, in the presence of 20 water molecules, is significantly influenced by the orientation of these molecules on the metallic surface, which is a key factor in determining their effectiveness. The adsorption energy value of NiFe_2_O_4_ (311) on the HKUST-1 surface in the existence of water molecules was obtained −4279 kcal mol^−1^ (−984.17 kJ mol^−1^) (Fig. 18a and b). The negative *E*_ads_ values, indicative of physical and favorable adsorption, confirmed the exothermic and spontaneous nature of the adsorption process of NiFe_2_O_4_ (311) onto the HKUST-1 surface.^63,64^

#### 3.8.2. The adsorption of GO compounds on NiFe_2_O_4_(311)@HKUST-1 and CR molecules on NiFe_2_O_4_(311)@HKUST-1/GO

**Fig. 19:**
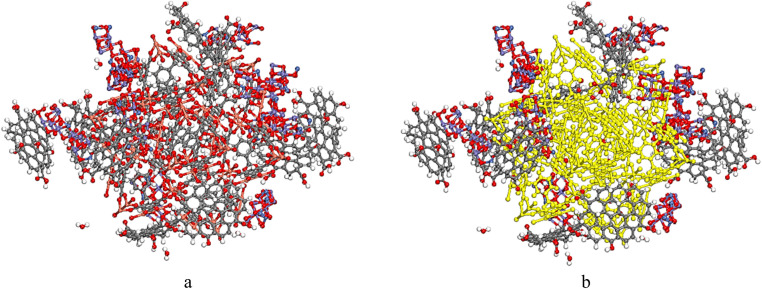
(a and b) The adsorption of GO on NiFe_2_O_4_(311)@HKUST-1 surface to form NiFe_2_O_4_(311)@HKUST-1/GO by Monte Carlo simulation.

**Fig. 20:**
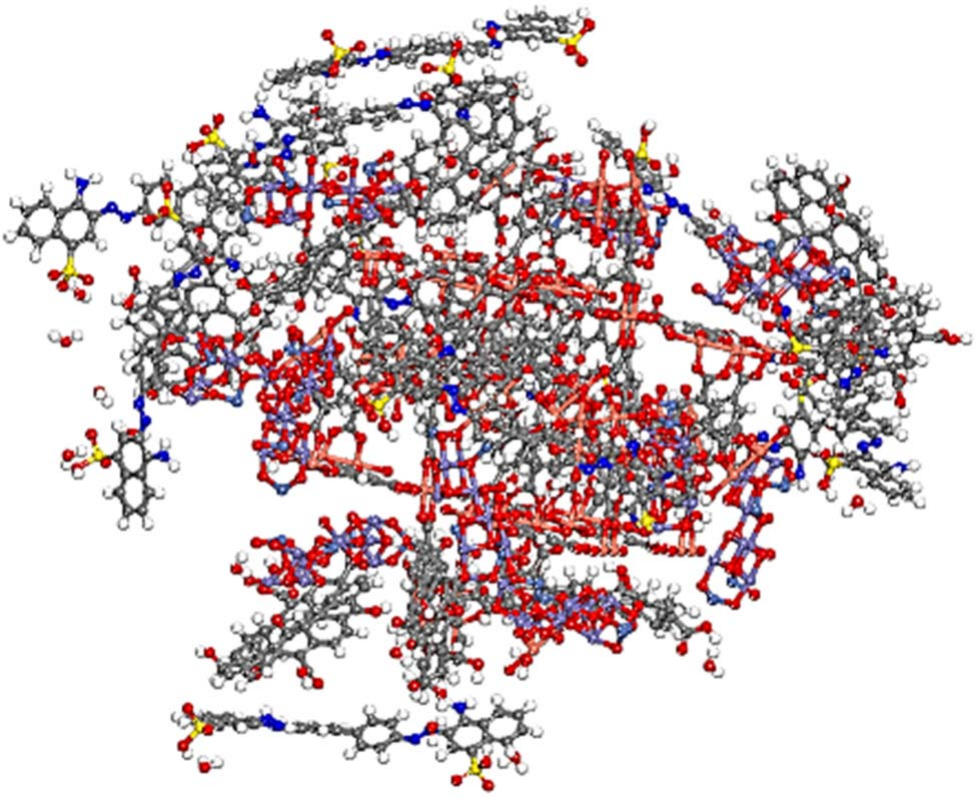
The lowest adsorption configuration of adsorption of CR molecules on NiFe_2_O_4_(311)@HKUST-1/GO by Monte Carlo simulation.

The adsorption energies of GO compounds on NiFe_2_O_4_(311)@HKUST-1 and CR molecules on NiFe_2_O_4_@HKUST-1/GO are −7651 kcal mol^−1^ (−1759.73 kJ mol^−1^) and −2259 kcal mol^−1^ (−519.57 kJ mol^−1^) in aqueous media, respectively. Therefore, GO and CR are the most strongly adsorbed on NiFe_2_O_4_(311)@HKUST-1 and NiFe_2_O_4_(311)@HKUST-1/GO, respectively. Additionally, the more stable adsorption configurations reveal that the distances between the molecules and the surfaces are less than 10 Å, indicating that the adsorption process is primarily physical in nature. This suggests that van der Waals forces and electrostatic interactions play a significant role in the adsorption, rather than stronger covalent or ionic bonds. A more negative value of adsorption energy (*E*_ads_) indicates stronger adsorption between GO and CR dye on the studied surface. This suggests that the GO and CR molecules are more favorably bound to the surface, with the adsorption process being both exothermic and spontaneous.^65^ A more negative *E*_ads_ value typically reflects a higher tendency for GO and CR to adhere to the surface, the equilibrium configurations are represented in Fig. 19a, b and 20.

Compounds interact with the vacant d-orbitals of iron atoms initially through physisorption by donating π-electrons and lone pairs from their aromatic and heterocyclic moieties. This interaction can progress to chemisorption, resulting in the formation of coordination bonds.^66,67^ Such bonding enhances the inhibition performance by effectively covering the metal surface, approximately matching the area of the compound's flat molecular projection.^66,67^

### 3.9. Novelty of studies

Although similar core–shell systems containing ferrites and graphene oxide have been reported, our study presents a distinct NiFe_2_O_4_@HKUST-1/GO nanocomposite synthesized through a layer-by-layer assembly approach. This design offers several key advancements. (A) This work represents a rare example of a composite that combines NiFe_2_O_4_ (a magnetic ferrite), HKUST-1 (a highly porous metal–organic framework), and GO (a high surface area support) into a unified core–shell hybrid structure. This integration significantly enhances surface functionality and provides improved adsorption pathways. (B) The nanocomposite exhibits a maximum adsorption capacity of 25.64 mg g^−1^, surpassing that of many individual or binary systems such as MOF/ferrite or MOF/GO under similar conditions. Notably, it maintains this performance at neutral pH, where many MOFs typically suffer from structural degradation. (C) Monte Carlo simulations provide new theoretical insight into the adsorption mechanism at the molecular level by modeling the interactions among. This computational–experimental correlation enhances our understanding of active binding sites and adsorption pathways, which has not been extensively addressed in similar studies.

## 4. Conclusion

A novel magnetic three-component nanocomposite consisting of HKUST-1, NiFe_2_O_4_, and graphene oxide was synthesized using a layer-by-layer assembly method and applied for the adsorption of Congo Red, an organic contaminant dye. The composite was thoroughly characterized using XRD, FTIR, EDS, FESEM, and VSM techniques. Key parameters influencing the adsorption process—including solution pH, contact time, initial dye concentration, and adsorbent dosage—were systematically investigated. The optimal conditions were identified as pH 7, a contact time of 60 minutes, an initial dye concentration of 5 ppm, and 0.05 g of adsorbent. Adsorption isotherm analysis revealed that the data fit well with both Langmuir and Freundlich models, indicating the involvement of both monolayer and heterogeneous surface adsorption. Regarding the adsorption kinetics and according to the calculations, we found that the adsorption process follows the second-order kinetics. The adsorption of NiFe_2_O_4_ (311) molecules on HKUST-1 surface to form NiFe_2_O_4_@HKUST-1, GO molecules on NiFe_2_O_4_@HKUST-1 to form NiFe_2_O_4_@HKUST-1/GO, and CR on NiFe_2_O_4_@HKUST-1/GO were carried by Monte Carlo simulation. The negative values of *E*_ads_ indicate high stability of the adsorptive system, signifying a strong interaction between the adsorbate and the surface. It also implies that the adsorption process is spontaneous, requiring no external energy input to proceed.

## Conflicts of interest

There are no conflicts to declare.

## Supplementary Material

RA-015-D5RA02381E-s001

RA-015-D5RA02381E-s002

RA-015-D5RA02381E-s003

RA-015-D5RA02381E-s004

RA-015-D5RA02381E-s005

RA-015-D5RA02381E-s006

RA-015-D5RA02381E-s007

RA-015-D5RA02381E-s008

## Data Availability

The authors confirm that the data supporting the findings of this study are available within this article. It should be noted that all the data here are original and have not been published anywhere before. The data that support the findings of this study are available on request from the corresponding author. The data are not publicly available due to restrictions, as they containing information that could compromise the privacy of research participants.
