# Supplementary material for: Experimental and Monte Carlo simulation study on a core–shell NiFe2O4@HKUST-1/graphene oxide nanocomposite for Congo Red adsorption
Source: RSC Adv. 2025 Jul 2;15(27):21240–56. doi: 10.1039/d5ra02381e (PMC12216895; doi:10.1039/d5ra02381e)
Supplement: RA-015-D5RA02381E-s008 [file RA-015-D5RA02381E-s008.pdf]

This journal is © The Royal Society of Chemistry 2025

**Table 1S.** EDS spectra of $NiFe_2O_4$

| Elt | Line | Int | Error | K | Kr | W% | A% | ZAF | Formula | Ox% | Pk/Bg | Class | LConf | HConf | Cat# |
|---|---|---|---|---|---|---|---|---|---|---|---|---|---|---|---|
| **O** | Ka | 1237.0 | 247.2473 | 0.4730 | 0.3887 | 50.91 | 78.50 | 0.7636 | | 0.00 | 1214.14 | A | 50.47 | 51.34 | 0.00 |
| **Fe** | Ka | 821.9 | 0.8459 | 0.4345 | 0.3571 | 40.17 | 17.75 | 0.8890 | | 0.00 | 32.14 | A | 39.75 | 40.59 | 0.00 |
| **Ni** | Ka | 166.7 | 0.8459 | 0.0926 | 0.0761 | 8.92 | 3.75 | 0.8525 | | 0.00 | 9.62 | A | 8.72 | 9.13 | 0.00 |
| | | | | 1.0000 | 0.8219 | 100.00 | 100.00 | | | 0.00 | | | | | 0.00 |

**Table 2S.** EDS spectra of $NiFe_2O_4$@HKUST-1/GO

| Elt | Line | Int | Error | K | Kr | W% | A% | ZAF | Formula | Ox% | Pk/Bg | Class | LConf | HConf | Cat# |
|---|---|---|---|---|---|---|---|---|---|---|---|---|---|---|---|
| **C** | Ka | 482.1 | 93.2414 | 0.2487 | 0.1163 | 31.36 | 44.15 | 0.3709 | | 0.00 | 962.36 | A | 30.94 | 31.79 | 0.00 |
| **O** | Ka | 679.0 | 106.1730 | 0.3560 | 0.1665 | 45.89 | 48.49 | 0.3628 | | 0.00 | 857.56 | A | 45.36 | 46.41 | 0.00 |
| **S** | Ka | 73.8 | 93.2312 | 0.0432 | 0.0202 | 2.38 | 1.26 | 0.8469 | | 0.00 | 9.02 | A | 2.30 | 2.47 | 0.00 |
| **Fe** | Ka | 397.5 | 0.6167 | 0.2881 | 0.1347 | 16.58 | 5.02 | 0.8125 | | 0.00 | 28.11 | A | 16.33 | 16.83 | 0.00 |
| **Ni** | Ka | 81.0 | 0.6167 | 0.0617 | 0.0289 | 3.64 | 1.05 | 0.7929 | | 0.00 | 8.56 | A | 3.52 | 3.76 | 0.00 |
| **Cu** | Ka | 3.0 | 0.6167 | 0.0023 | 0.0011 | 0.14 | 0.04 | 0.7515 | | 0.00 | 2.28 | B | 0.12 | 0.17 | 0.00 |
| | | | | 1.0000 | 0.4677 | 100.00 | 100.00 | | | 0.00 | | | | | 0.00 |
